# Assessing the empathy of medical students during medical interview training in Japan by using mixed-methods surveys of simulated patients

**DOI:** 10.15694/mep.2019.000179.1

**Published:** 2019-09-17

**Authors:** Yoshimi Harada, Yoji Hirayama, Kana Wakuda, Hiromi Imura, Junji Otaki

**Affiliations:** 1 Department of General Medicine and Primary Care

**Keywords:** medical interview, empathy, OSCE, simulated patients, nonverbal communication, medical student

## Abstract

This article was migrated. The article was marked as recommended.

**Background:** Medical interviews are performed during objective structured clinical examinations, to assess the communication skills of medical students. In particular, medical students are assessed regarding whether they demonstrate empathy during these staged interviews. However, no studies to date have analyzed the validity of the methods to evaluate empathy during objective structured clinical examinations.

**Aim:** Here we sought to identify factors affecting whether simulated (standardized) patients (SPs) felt that the medical students had treated them empathetically during medical interview training.

**Methods:** The training involved the participation of SPs during the bedside learning of fifth-year medical students in Japan. After the students completed medical interview training, we conducted a questionnaire-based survey. We developed the list of questionnaire items through semi-structured interviews asking SPs to describe when the student had been empathetic.

**Results:** The item “I felt that the student was empathic throughout the interview” was significantly correlated with “I was given enough time to talk at the beginning of the interview,” “The student made good eye contact,” “The student’s attitude was appropriate,” “I was able to say enough of what I wanted to talk about,” and “The student used phrases that expressed empathy.” Multiple regression analysis revealed that “The student used phrases that expressed empathy” was the only independent predictor of “I felt that the student was empathic throughout the interview.”

**Conclusions:** The factor that correlated most strongly with the SP feeling that the student was empathic during a medical interview was “The student used phrases that expressed empathy.” However, correlations also occurred with open-ended questions and the student’s level of attention at the beginning of the interview and with the student’s attitude. Together, these findings indicate that several types of both verbal and nonverbal communication determined whether SPs felt that medical students showed empathy during staged medical interviews.

## Introduction

The skill and knowledge required of doctors are rapidly increasing, and training in professionalism is regarded an important aspect of becoming a doctor. One of the foundations supporting this professionalism is communication skills ( Stern, 2006). An attitude of empathy that endeavors to understand things from the patient’s viewpoint is particularly important in communication in healthcare, and efforts to foster this attitude are being incorporated into medical education ( Pedersen, 2010).

The primary context for communication between doctors and their patients is the medical interview, and role-playing with simulated (i.e., standardized) patients (SPs) is an effective technique for training and evaluation ( Rees, Sheard and McPherson, 2002; Chan *et al*., 2003; Yedidia *et al*., 2003). The development of an empathetic attitude is one aspect of medical education, and empathy training bridges the gap between biomedicine and the humanities ( Pedersen, 2010). However, although diverse methods have been used to investigate empathy, no consistently informative measure (‘gold standard’) is available ( Eikeland *et al*., 2014).

Both Japanese and overseas studies have demonstrated gender-associated differences in doctors’ communication abilities and styles. For example, female doctors typically exhibit greater empathy than do male doctors ( Roter and Hall, 2004; Swygert *et al*., 2012; Shin *et al*., 2015; Vogel, Meyer and Harendza, 2018). However, targeted medical interview training throughout medical school may help decrease this gender-associated gap ( Harada *et al*., 2018).

Objective structured clinical examinations (OSCEs) are widely used to evaluate medical students’ acquisition of proficiencies that are difficult to assess through written or oral tests, including decision-making, clinical skills, bedside manner, and medical interviewing ( Harden *et al*., 2016). In most countries, OSCEs are included in the examinations for graduation or for national medical licensure. In an OSCE, whether the examination candidate displayed an empathetic attitude toward the SP is gauged by a third-party assessor, who applies various defined criteria, chiefly whether the candidate used empathetic phrases when talking with patients. However, a study of evaluations by two different assessors ( Kubota *et al*., 2009) revealed numerous inconsistencies between their evaluations of “listening” and “empathy.” The basis for empathy evaluation in the OSCE should not rest solely on arbitrary judgments by an assessor, and specific parameters for observation during OSCEs should be identified to develop a more objective structure for evaluation. Several scales have been developed for the evaluation of communication, including conveying empathy, in medical interviews. The best known of these, the Kalamazoo consensus statement ( Makoul, 2001), was written by a panel of experts. Other scales have similarly been established on the basis of specialist opinions ( Mercer *et al*., 2004; Humphris and Kaney, 2001) or literature reviews ( Kane *et al*., 2007; Kurtz and Silverman, 1996; Huntley *et al*., 2012; Ishikawa *et al*., 2006). However, none of these previous studies describe the development of a patient-centered scale. The objective of the current study was to identify factors that influenced whether trained simulated (i.e., standardized) patients (SPs) felt that they had been treated empathetically during medical interviews conducted by medical students.

## Methods

### 1. Development of questionnaire

We had developed the questionnaire through semi-structured interviews as follows asking SPs to describe when the student had been empathetic ( Harada *et al*., 2018).

#### 1-1. Subjects

In Japan, undergraduate medical education is six years long. Fifth-year Japanese medical students undergoing clinical training in the Department of Cardiology of Tokyo Medical University took part in medical interview practice with SPs. Standard signs and diseases not limited to cardiology were included in the scenarios used. Role-playing during training interviews was done in groups of 7 or 8 people, with 2 students in each group taking the doctor’s role. The study subjects were 18 individual SPs, who participated in 34 SP interviews with 34 students in 17 groups between 30 March 2015 and 16 February 2016.

#### 1-2. Semi-structured interviews

After the role-playing sessions, each SP was asked (1) whether the student had been empathetic (i.e., had treated the SP empathetically) during the medical interview, and (2) at what points the SP felt that the student had displayed empathy.

#### 1-3. Analysis and questionnaire development

The audio data from the interviews were transcribed, coded, and categorized according to the standard method of content analysis. A questionnaire was developed that asked about each of the items on a 5-point Likert scale, with responses ranging from 0 to 4 (Supplementary File 1).

### 2. Questionnaire-based survey

#### 2-1. Subjects

The subjects of the survey were 36 SPs who participated in practice interviews with a total of 36 fifth-year medical students. Role-playing and training were done in 18 groups between 12 April 2016 and 14 February 2017.

#### 2-2. Survey process

After the training sessions had been completed, the developed questionnaire (Section 1-3) was distributed to the SPs, with the request to complete it within 2 weeks. After 2 weeks, we collected all completed questionnaires.

#### 2-3. Analysis

The reliability of the questionnaire was investigated by using Cronbach’s alpha coefficient, and Spearman’s correlation coefficient was calculated to assess the association between each item and the statement “I felt that the student was empathetic throughout the interview.” Multiple regression analysis was performed also. Statistical analysis was performed by using IBM SPSS Statistics 24, and a *P* value less than 0.05 was regarded as significant.

## Results/Analysis

### 1. Development of questionnaire

During fifth-year training in the 2015 academic year, 34 Japanese students participated in medical interviews with 18 individual SPs in a total of 34 SP interviews. During semi-structured interviews the SPs responded that “I felt that the student was empathetic throughout the interview” for 13 (38%) of those 34 interviews. The SPs’ impressions regarding the statement that “I felt that the student was empathic throughout the interview” were classified into the following categories: “I was given enough time to talk at the beginning of the interview,” “The volume and clarity of the student’s voice were appropriate,” “The student made good eye contact,” “The student’s attitude was appropriate,” “I was able to say enough of what I wanted to talk about,” and “The student used phrases that expressed empathy.”

### 2. Questionnaire-based survey

**Figure 1.**
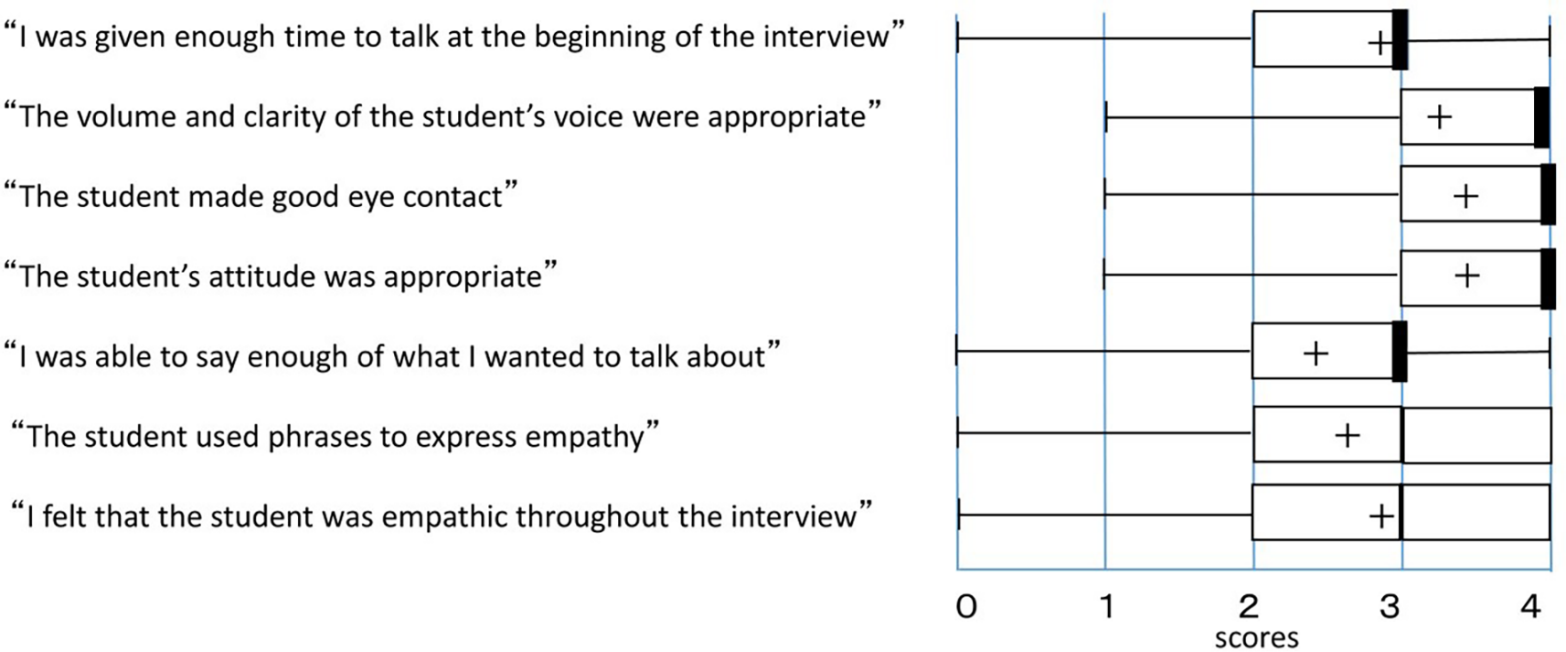
Results of questionnaire survey - Contents of question items and evaluation from simulated patients (SPs).

Responses were obtained from 36 SPs who had participated in staged medical interviews with 36 medical students. The students received high scores overall, with a few students receiving low scores for some items. The median scores and interquartile ranges for each questionnaire item are shown in Figure 1.

**Figure 2.**
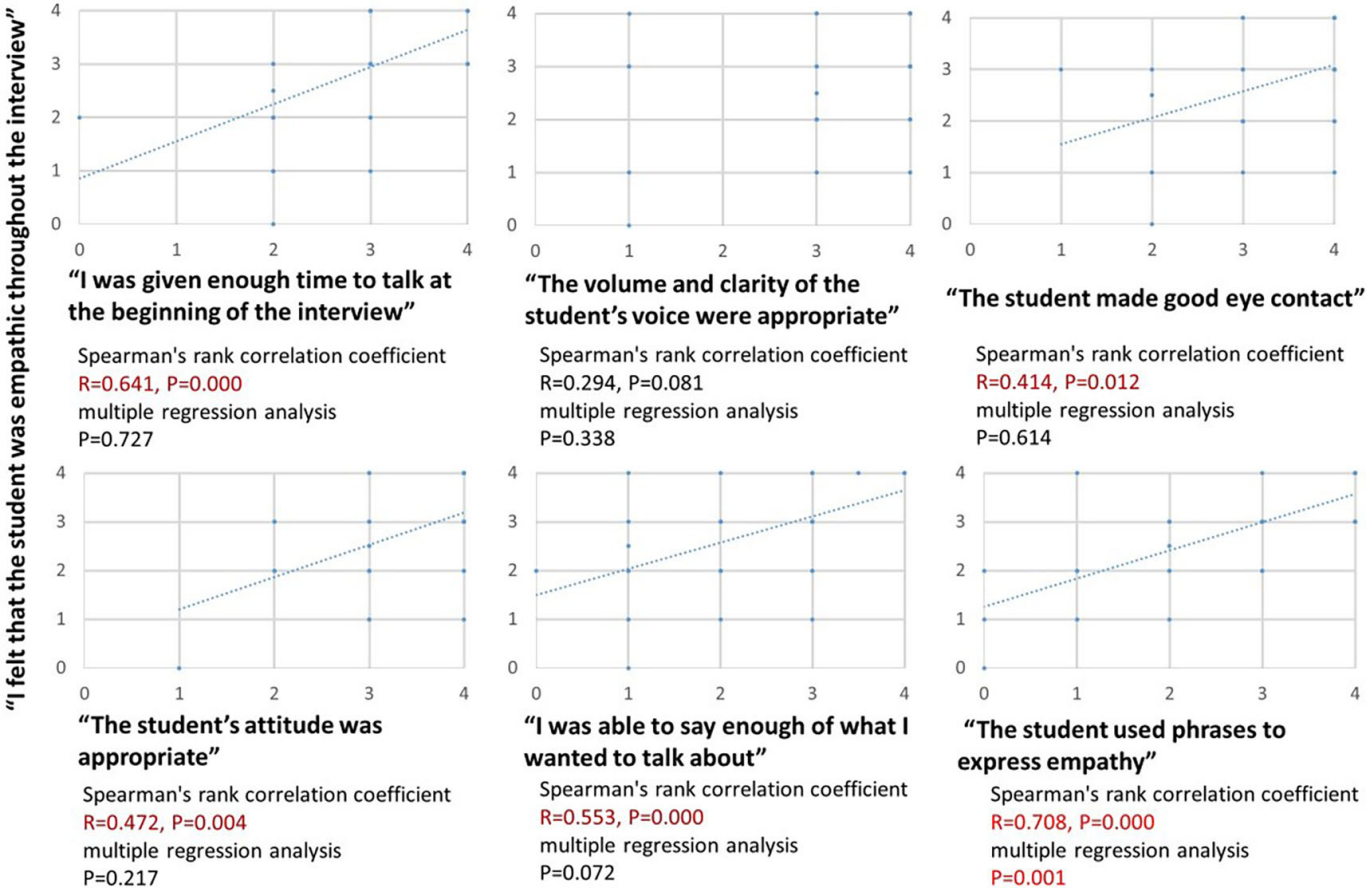
Results of questionnaire survey - Correlation between each question item and empathy.

Multiple regression analysis demonstrated that “The student used phrases to express empathy” was the only independent predictor of “I felt that the student was empathic throughout the interview” ( *P* < 0.001) ( Figure 2).

Cronbach’s alpha coefficient (0.827) showed good internal consistency.

## Discussion

We investigated factors from the patient (SP) perspective that influenced whether SPs felt that fifth-year medical students in Japan wereempathetic during staged medical interviews. Multiple regression analysis demonstrated that “The student used phrases to express empathy” was the only independent predictor of “I felt that the student was empathic throughout the interview.” However, in some interviews, the SPs felt that the students had displayed empathy despite not using empathetic phrases. This finding suggests that empathy cannot be evaluated merely by measuring the extent to which it is communicated verbally. Likewise, during the semi-structured interviews that were used to produce the questionnaire, SPs mentioned various additional nonverbal expressions through which they felt that a student displayed empathy, including “I was given enough time to talk at the beginning of the interview” and “The student made good eye contact.”

Pedersen (2010) reported that as medical students go through their educational program, their frequency to empathize decreases, and in the later years of training, their interest tends to focus more intently on the scientific aspects of medicine. Given that fifth-year medical interview training was conducted during clinical training, students in this study may have been biased toward soliciting medical-scientific information, or they may have experienced the fact that sufficiently empathetic medical interviews are not typically conducted in clinical practice ( Swygert *et al*., 2012). Eikeland *et al*. (2014) reported that the hidden curriculum of actual clinical practice inhibits empathy.

Diverse methods have been used to investigate empathy, but no consistently informative measure (‘gold standard’) has emerged yet ( Eikeland *et al*., 2014). Scales for objectively evaluating the empathy of medical students ( Hojat *et al*., 2001) and parameters for assessing nonverbal communication in medical interviews ( Sugawara *et al*., 2017) have been proposed. The consultation and relational empathy measure (CARE) has been developed as a scale for the qualitative and quantitative measurement of empathy in actual clinical settings ( Mercer *et al*., 2004). The reliability of CARE has been investigated in Japanese populations as well as in predominantly white populations in the United Kingdom ( Matsuhisa *et al*., 2018). However, CARE includes items regarding explanations and treatments, thereby making it unsuitable for evaluating student medical interviews, and its content is somewhat abstract. The proposed Jefferson Scale of Patient’s Perceptions of Physician Empathy (JSPPPE) was developed on the basis of a literature review. JSPPPE asks patients to identify points at which they perceived empathy during medical interviews ( Kane, 2007) and measures five factors that primarily address verbal communication, emphasizing the use of phrases to communicate empathy in its evaluation. On the other hand, we couldn’t find any report of study of staged medical interviews performed by students during their early training.

Strong nonverbal communication skills are reportedly associated with high scores regarding patient satisfaction ( Griffith *et al*., 2003 Ishikawa *et al*., 2010). Ishikawa *et al*. (2006) used previous studies to compile a list of parameters for evaluating nonverbal communication. Those previous studies show it is insufficient to evaluate empathy in medical interviews solely on the basis of the observation by the assessor with phrases expressing empathy. The results of our semi-structured interviews suggest that the evaluation of empathy must incorporate measures of nonverbal as well as verbal communication. In the current study, we developed questionnaire items by analyzing the impressions of SPs who participated in medical interview training. As Huntley *et al*. (2012) have noted, some aspects of the quality of communication cannot be assessed by an observer (third party), and evaluation by SPs may be useful in terms of improving validity and providing feedback to student trainees. In addition, the content of the items we developed may be useful for future studies of empathy evaluation. Communication skills training programs are effective in raising students’ awareness in the short term but have little long-term effect ( Kataoka *et al*., 2018) and are reportedly insufficient to change actual performance ( Ishikawa *et al*., 2010). Repeated training with the content that we identified in the current study may be effective to achieve long-term effectiveness.

The questionnaire used in this study was judged to be valid because it was developed by using categories identified from semi-structured interview data. In terms of reliability, Cronbach’s alpha was 0.827, indicating acceptable internal consistency. A limitation of our study is its nature as a single-center study of SPs belonging to a single organization. Because the diversity of both the participants and their backgrounds was thus narrow, evidence supporting the results as generalizable may be insufficient. In addition, multiple scenarios were used during the training, and different effects of these scenarios cannot be ruled out. Further multicenter studies of medical students at different stages of their education with the participation of either SPs from several organizations or actual patients are warranted.

## Conclusion

The factor that correlated most strongly with whether SPs felt that Japanese fifth-year medical students were empathic during medical interview training was “The student used phrases to express empathy.” However, correlations were observed with open-ended questions and the student’s level of attention at the beginning of the interview as well as the student’s attitude in general. Together, our current findings indicate that various types of both verbal and nonverbal communication determined whether SPs felt that medical students showed empathy during staged medical interviews.

## Take Home Messages

• The factor that correlated most strongly with whether simulated (standardized) patients felt that students were empathic during medical interview training was “The student used phrases to express empathy.”
• Evaluation of empathy must incorporate various aspects of both verbal and nonverbal communication.
• SPs may be helpful for improving validity and providing feedback to student trainees.

## Notes On Contributors

**Yoshimi Harada, MD, PhD** is an Associate Professor, Department of General Medicine and Primary Care, Tokyo Medical University, Tokyo, Japan.

**Yoji Hirayama, MD, PhD,** is a Professor, Department of General Medicine and Primary Care, Tokyo Medical University, Tokyo, Japan.

**Kana Wakuda, BA,** is an Office Administrator, Department of General Medicine and Primary Care, Tokyo Medical University, Tokyo, Japan.

**Hiromi Imura, MD, PhD,** is an Adjunct Instructor, Department of General Medicine and Primary Care, Tokyo Medical University, Tokyo, Japan.

**Junji Otaki, MD, DMEDSC,** is an Adjunct Professor, Department of General Medicine and Primary Care, Tokyo Medical University, Tokyo, Japan.
