# Supplementary material for: Assessing the empathy of medical students during medical interview training in Japan by using mixed-methods surveys of simulated patients
Source: MedEdPublish (2016). 2019 Sep 17;8:179. [Version 1] doi: 10.15694/mep.2019.000179.1 (PMC10712511; doi:10.15694/mep.2019.000179.1)
Supplement: Peer review history

# Reviewer report: Reviewer response for version 1

**DOI:** 10.21956/mep.19912.r30167

**Published:** 2019-09-25

**Authors:** Trevor Gibbs

**Affiliations:** 1 AMEE

This review has been migrated. The reviewer awarded 4 stars out of 5 An interesting paper covering a very important topic. The authors are quite right in their review of the literature on the subject of measuring empathy and this approach has similarity between it and the use of simulated patients to globally rate the students during an OSCE.I felt that the paper was well researched, their methodology was sound, but as frequently observed, would have derived more benefit from more participants over a more diverse population.Like my co-reviewer , I agree that the paper could have been improved by outlining its limitations and how the authors would take this research forward - from OSCE to reality.I would recommend this paper to all those involved in the development and assessment of communications skills , including the subject of empathy.

Reviewer Expertise:

NA

No decision status is available

# Reviewer report: Reviewer response for version 1

**DOI:** 10.21956/mep.19912.r30169

**Published:** 2019-09-18

**Authors:** Subha Ramani

**Affiliations:** 1 Harvard Medical School, Brigham and Women's Hospital

This review has been migrated. The reviewer awarded 4 stars out of 5 The authors have conducted a study on one of the most fundamental clinical skills, communication skills, specifically focusing on defining empathy. Their exploration of this entity from the patient's perspective is mot appropriate.They used good study design to answer their study questions, performed a mixed method approach to tease out what patients believed are elements of an empathic interaction, and developed a questionnaire study which was checked for validity and reliability. I agree that a questionnaire on empathy derived from patients indicated validity of the data obtained.Their conclusions are commensurate with their results.A couple of things I would have liked to have seen under discussion: limitations of this study, next steps as well as future areas for research. For eg: I would like to see an extension of this inquiry into real patient encounters. Some more narratives from patients would further enrich the data.All educators interested in teaching and studying communication skills / empathy will find this paper useful.

Reviewer Expertise:

NA

No decision status is available

# Reviewer report: Reviewer response for version 1

**DOI:** 10.21956/mep.19912.r30168

**Published:** 2019-09-17

**Authors:** Felix Silwimba

**Affiliations:** 1 University of Lusaka

This review has been migrated. The reviewer awarded 5 stars out of 5 this study is very useful . it has helped me understand the role of standardized patients in OSCE. it is applicable everywhere.

Reviewer Expertise:

NA

No decision status is available
